# Effect of Transportation Time on Weaner Pigs’ Welfare and Productive Losses in a Semi-Arid Region

**DOI:** 10.3390/vetsci12030214

**Published:** 2025-03-01

**Authors:** Ana Letícia Vieira e Silva, Nítalo André Farias Machado, José Antonio Delfino Barbosa-Filho, Carla Renata Figueiredo Gadelha, Jordânio Inácio Marques, Patrício Gomes Leite, Andressa Carvalho de Sousa, Wellington Cruz Corrêa, Maria Gabriela Marcineiro Araújo, Andreza Maciel de Sousa, Telmo José Mendes, Marcos Vinícius da Silva

**Affiliations:** 1 Centro de Ciências Chapadinha, Universidade Federal de Maranhão, Chapadinha 65500-000, MA, Brazil; ana.ads@discente.ufma.br (A.L.V.e.S.); nitalo.farias@ufma.br (N.A.F.M.); jordanio.marques@ufma.br (J.I.M.); patricioo.leite@ufma.br (P.G.L.); andressa.carvalho1@discente.ufma.br (A.C.d.S.); wellington.cruz@discente.ufma.br (W.C.C.); maria.marcineiro@discente.ufma.br (M.G.M.A.); andreza.maciel@discente.ufma.br (A.M.d.S.); telmo.mendes@ufma.br (T.J.M.); 2 Centro de Ciências Agrárias, Universidade Federal do Ceará, Mister Hull, Fortaleza 60440-554, CE, Brazil; zkdelfino@ufc.br (J.A.D.B.-F.); crgadelha@yahoo.com.br (C.R.F.G.)

**Keywords:** animal transport, stress, thermoregulation, productive losses

## Abstract

Reducing losses during pig transport is essential for breeders and transporters, particularly in semi-arid regions, where high temperatures exacerbate transport-related stress and risk of losses. This study aimed to evaluate the effect of transport duration (short vs. long trips) on animal welfare and production losses during the commercial transport of weaner pigs in a semi-arid region. A total of 20 commercial journeys were monitored, with transport times of 30 min (15 km) and 150 min (170 km). Upon arrival, physiological and behavioral stress indicators were assessed in 960 weaner pigs (26.4 ± 2.8 kg body weight, 48 per journey). Production losses were determined by calculating the percentage of injured pigs (NAI), fatigued pigs (NANI), and those that were dead on arrival (DOA), whereas the total loss was expressed by the sum of NAI + NANI + DOA. Weaner pigs transported for 30 min exhibited significantly higher (*p* < 0.05) rectal temperature, respiratory rate, and stress biomarkers (cortisol and creatine kinase levels). Additionally, this group showed a higher percentage (*p* < 0.05) of “sitting” pigs and a lower percentage of “lying” pigs in transit, as well as a higher frequency of agonistic behavior after transport compared to those transported for 150 min. Furthermore, higher production losses were recorded in the 30 min transport group, primarily due to the increased percentage of fatigued pigs and DOA pigs. Therefore, shorter transport operations in the Brazilian semi-arid region increased the risk to animal welfare and productive losses, likely due to handling-induced stress during loading.

## Simple Summary

Transporting pigs can be highly stressful for the animals, particularly in hot and dry climates where environmental conditions exacerbate their discomfort. This study examined how transport time impacts the health and welfare of weaner pigs. We monitored 20 commercial journeys in a semi-arid region, comparing short trips of 30 min with longer trips of 150 min. Upon arrival, we assessed the animals’ stress levels, estimated production losses, and evaluated their behavioral responses. Our findings indicate that pigs subjected to shorter transport durations exhibited higher stress levels and greater losses, including fatigue and mortality, compared to those transported for longer durations. This information is vital for farmers, transporters, and policymakers aiming to improve animal care and reduce economic losses during pig transportation.

## 1. Introduction

Animal transportation represents a critical risk to their welfare and survival, as well as potential economic losses for the livestock industry. During the road transportation of pigs, common issues include injuries, bruises, in-transit mortality, and the development of undesirable characteristics in meat and carcass quality [1]. According to the Scientific Opinion of the European Food Safety Authority [2], this fact has led to the implementation of numerous studies on transportation conditions within pig production systems. However, research addressing the transportation of weaned pigs (10–40 kg) remains scarce [3], particularly in semi-arid and tropical regions [4].

Given the limited thermoregulatory capacity of pigs due to their reduced sweating ability, transport conditions can significantly impact animal welfare and productivity [5]. However, the road transport of pigs occurs globally under a wide range of environmental and climatic conditions, varying by transport distance, road type, stocking density, vehicle design, time of travel, and country-specific regulations [6,7].

Sutherland et al. [8] reported that the mortality rate (DOA) of pigs upon arrival was significantly increased when external temperatures exceeded 25 °C. Averós et al. [9] investigated the DOA rate of weaned piglets during transportation, analyzing 58,682 piglets across 109 trips in Europe. The DOA rate was 0.07%, occurring in 13.8% of the loads. Both trip duration (0.3 to 69 h) and external temperatures (0 °C to 38 °C) contributed to increased mortality rates. Previous studies also indicated that weaned pigs may experience weight loss due to dehydration and reduced feed intake after transport, with in-transit deaths linked to hypothermia in certain conditions [10,11].

The combination of high temperatures, vehicle design limitations, and the quality of loading/unloading management are critical factors affecting the efficiency and welfare outcomes of transport operations [5,7]. Understanding how transport duration interacts with these variables is essential for optimizing transport protocols and mitigating stress in challenging climatic conditions. We hypothesize that both short and long transport durations impose distinct stressors on animals. During shorter trips, weaner pigs may not have sufficient time to recover from or adapt to the stress induced by physical exertion during loading and sudden environmental changes (e.g., mixed loads, feed and water deprivation, heat stress, vibrations, and noise, etc.), particularly under high thermal loads.

Most vehicles used for pig transportation in tropical and semi-arid conditions lack temperature control, exposing animals to potential heat stress [12]. These conditions further increase risks to pig welfare and economic losses [6]. Thus, the transportation of weaned pigs to finishing farms using segregated weaning management systems is a critical issue, particularly given the need to assess animal welfare risks and transport profitability in semi-arid climates.

This study aimed to evaluate the effect of transportation time (30 vs. 150 min) on physiological indicators, animal behavior, and productivity losses during weaner pig transportation in a Brazilian semi-arid region.

## 2. Materials and Methods

### 2.1. Animals and Farm and Transport Conditions

The study was conducted following ethical standards for animal research, after approval by the Animal Use Ethics Committee of the Agrarian Sciences Center at the Federal University of Ceará (Protocol number 9871250719). A total of 1920 weaned pigs of commercial lineage (Topigs Norsvin), approximately 68 days old, with an average body weight of 25.2 ± 5.3 kg, were transported during the summer of 2019 (January to February) in the state of Ceará, northeastern Brazil. The transportation routes included a farm in Maracanaú (3°54’46.4” S, 38°39’19.2” W, 43 m altitude) to finishing farms located in Morada Nova (5°06’24″ S, 38°22’21″ W, 52 m altitude), involving a 150 min (~170 km) trip, and Caucaia (3°44′4″ S, 38°39′23″ W, 24 m altitude), involving a 30 min (~15 km) trip. The transport operations commenced in the afternoon (15:00 to 17:00), and in some cases, journeys extended into the night. The transports were conducted by trained drivers from the animal transport company on paved federal highways. The selected transport times reflect common commercial practices in the region. Transport was conducted using a 13-ton capacity truck equipped with a two-tiered trailer. Each tier consisted of six compartments with dimensions of 0.95 m height, 2.40 m length, and 1.30 m width, totaling an area of 3.20 m^2^ per compartment. More details on the truck model and trailer used in this study can be found in previous studies [4,6], which provide comprehensive descriptions and images of the transport vehicle configuration. As a standard practice in Northeast Brazil in 2019, the vehicle was not equipped with air conditioning or environmental control systems, and the compartments lacked bedding and a drinking water supply. National regulations on animal transport came into effect in 2021 [13], and this study was conducted before their enforcement. The transport stocking density was 290 kg/m^2^, which is within the range used in commercial pig transportation in the study region, but exceeds the European Union recommendation of 235 kg/m^2^, which aims to minimize heat stress and maximize space for rest. Higher stocking densities may increase heat dissipation challenges and limit pigs’ ability to thermoregulate by adopting postures that facilitate heat loss. The feeding of the animals was suspended 120 ± 15 min before the start of the transport operation. The transport operations followed the Load-Cooling Through Showering protocol adopted by the company. This protocol consists of wetting the trailer before, during, and after the loading of the piglets. A designated employee used a hose to randomly spray water inside the trailer, ensuring that the cooling process began even before the animals entered. The duration of this procedure varied but consistently exceeded the time required for loading. To maintain control over water usage, the total volume applied during each journey was recorded, averaging 2500 ± 120 L per journey. During loading, all pigs were guided using flags to minimize excessive agitation and stress. Pre-fabricated concrete ramps with a 30° incline were used for loading the lower deck, while a 3 m metal ramp with a 58° incline was used for loading the upper deck.

### 2.2. Physiological Indicators

In this study, physiological indicators were measured as validated physiological markers of transport-induced stress, allowing for a comprehensive assessment of thermal and handling stressors on animal welfare. A subsample of 960 sentinel weaned pigs (480 per treatment; 48 per trip; 4 per compartment of the trailer; body weight: 26.4 ± 2.8 kg) was randomly selected for the physiological assessments. Selection occurred as the animals were guided toward the trailer, ensuring that the marked pigs were evenly distributed across compartments. To facilitate identification upon arrival, each selected pig was marked with non-toxic paint on the dorsal region during handling, before entering its assigned compartment. This marking procedure aimed to minimize handling stress, reduce the potential compartment effect, and ensure consistency in data collection [4]. The collection of rectal temperature (°C), surface temperature (°C), respiratory rate (RR, breaths/minute), as well as samples for the estimation of serum lactate concentration (m/M) and creatine kinase (CK, U/L), was performed approximately 25 ± 5 min after transportation (arrival at the final destination). Before handling, the respiratory rate and surface temperature were measured. The respiratory rate was obtained by observing flank movements for 30 s. Surface temperature was measured using a thermographic camera with 4800-pixel resolution (Fluke, TiS10, Everett, WA, USA), as described by Machado et al. [4]. The animals were then briefly restrained in dorsal recumbency for approximately 2 min to collect blood samples from the jugular vein. Ten milliliters of blood were collected in tubes (BD Vacutainers, Kasvi K50204S, São José dos Pinhais, Brazil). For cortisol analysis, a commercial ELISA kit (Neogen Corp., Lexington, KY, USA) was used with a microplate reader. Creatine kinase concentration was determined using a commercial kit (Creatine Kinase-SL, Sukisui Diagnostics, Charlottetown, PE, Canada) and measured using a spectrophotometer (Konica Minolta, CM-3600A, Tokyo, Japan).

### 2.3. Animal Behavior

In addition to physiological indicators, we carry out an assessment of animal behavior. During transportation, the behavior of the weaned pigs was continuously recorded using four cameras (Intelbras VMH 1010 D HD 720p, Intelbras SA, São José, Brazil) strategically positioned at each corner of the trailer. A trained observer analyzed the recordings, counting the number of pigs in each posture (lying, standing, sitting, or any other posture) every 5 min, according to the ethogram adapted from Fox et al. [14], as shown in Table 1. For analysis, only recordings with at least 7 animals fully and clearly visible were considered. At the finishing barn, animal behavior was recorded during the first hour of rest post transportation by five trained observers using the scan sampling method [15,16]. Observations were conducted in 10 min intervals, specifically recording drinking and agonistic behaviors. To ensure reliability between observers, a standardized ethogram was used. The recorded frequencies were subsequently analyzed to assess post-transportation behavioral patterns.

### 2.4. Assessment of Production Losses

For evaluating production losses, parameters commonly used in pig slaughterhouses upon arrival were employed [17]. Observed categories of compromised pigs, such as non-ambulatory non-injured (NANI) and non-ambulatory injured (NAI), as well as the incidence of dead-on-arrival (DOA) pigs, served as critical indicators for this analysis. These indicators help evaluate and identify handling issues on the farm, during transport preparation, and transportation conditions (travel time, available space, and climatic control within the truck [18,19]. In this study, during unloading, an observer recorded the number of pigs classified as fatigued (NANI)—panting animals or those refusing to stand without apparent injuries, trauma, or disease; injured (NAI)—injured animals unable to move or with compromised mobility; and DOA pigs, according to Ritter et al. [20]. Using these parameters, total productive loss was estimated by summing the incidence of NANI, NAI, and DOA pigs, as proposed by [21].

### 2.5. Thermal Assessment

A total of 12 sensors (Onset, U23-001 HOBO Pro v2, Los Angeles, CA, USA) were installed at the center of each compartment of the truck trailer, at animal height. These sensors were programmed to record air temperature (°C) and relative humidity (%) every 10 min throughout the entire transport operation. The sensors remained in place until the conclusion of the experiment, ensuring continuous and reliable data collection. To contextualize the recorded internal conditions, transport departure and arrival times were documented, allowing for the assessment of micrometeorological conditions inside the truck. This monitoring enabled the characterization of the micrometeorological profile through specificity [22], calculated using Equation (1). According to data from meteorological stations of the National Institute of Meteorology of Brazil (INMET), solar radiation ranged from 450 to 510 kJ/m^2^, ambient temperature varied between 29.4 and 34.6 °C, and relative humidity ranged from 60 to 69% during the journeys.

(1) $$H=1.006\times AT+\frac{RH}{Pb}\times 10^{7.5TA{\left(237.3+TA\right)}^{-1}}\times (71.28+0.052\times TA)$$

where *H* is specific enthalpy (kJ/kg of dry air); *TA* is air temperature (°C); *RH* is relative humidity (%); and *Pb* is local barometric pressure (mmHg).

### 2.6. Statistical Analysis

Two groups of animals transported for 30 min and 150 min were compared. Data from Thermal Assessment, Assessment of Production Losses, and Physiological Indicators were analyzed using the MIXED procedure in the Statistical Analysis System (SAS Inst. Inc., Cary, NC, USA, version 3.3), based on the statistical model: Yij = μ + Ti + βXij + eij, where Yij = observed value; µ = overall mean; Ti = effect of transport time; βXij = effect of the collection week; and eij = effect of experimental error. The MIXED procedure was chosen as it accounts for both fixed and random effects, allows for the inclusion of repeated measures, and provides a robust framework for handling unbalanced data while improving statistical accuracy. Animal behavior data were analyzed using generalized linear mixed models for repeated measures with the GLIMMIX procedure in SAS, as the residuals did not follow a normal distribution. The assumption of normality was assessed using the Shapiro–Wilk test. For all variables, means were compared using the Tukey–Kramer test, accounting for variations in sample size due to factors such as contusions, mortality, and other unforeseen losses. A significance level of *p* < 0.05 was adopted for all tests.

## 3. Results

The mean values of the thermal assessment of the load are presented in Table 2. It was observed that relative humidity and enthalpy were affected (*p* < 0.01) by transport time in this study, with average increases of 7% in humidity and 3.75 kJ/kg of dry air in specific enthalpy for the load transported for 30 min. However, both transport conditions (i.e., 30 min vs. 150 min) exhibited mean enthalpy values above 80 kJ/kg of dry air, characterizing an environment conducive to “heat stress” in weaned pigs [23].

Transport time had an effect on all evaluated physiological variables, except for surface temperature. Increases in rectal temperature (+0.77 °C; *p* = 0.019) and respiratory rate (+8 breaths/min; *p* = 0.017) were recorded in the group of animals transported for 30 min. Additionally, higher concentrations of cortisol (+3.96 ng/mL) and creatine kinase (+684 U/L) were observed in the group transported for 30 min (both *p* < 0.001) compared to the group transported for 150 min, as shown in Table 3.

During transportation, the group transported for 30 min exhibited a higher percentage of weaned pigs in a sitting posture and a lower percentage in a lying posture compared to the group transported for 150 min (Table 4). Observations post transportation showed that transport time did not influence the animals’ postures but did affect the drinking behavior of the weaned pigs. A higher frequency (*p* = 0.018) of drinking behavior (+4.2%) was detected in animals transported for 30 min. Furthermore, a higher frequency of agonistic behaviors (+1.40%; *p* = 0.043) was observed in animals transported for 30 min (Table 4).

Regarding the proportions of productive losses in the load, an increase in the incidence of fatigued animals (+3.06%; *p* < 0.001) and mortality rates upon arrival (+4.08%; *p* = 0.027) was observed in the group transported for 30 min. This influenced the percentage of total productive loss, which significantly increased (+9.18%) in loads transported for 30 min compared to the group transported for 150 min (Table 5).

## 4. Discussion

One of the main concerns related to animal welfare in pig production is transportation [7], especially under challenging climatic conditions such as those found in semi-arid regions [6]. A semi-arid climate region is characterized by a high heat load and risk of heat stress, as confirmed by the thermal assessment results of the load in this study. Although transport time significantly influenced the relative humidity and enthalpy of the load, with higher values observed in loads transported for 30 min, the mean enthalpy values across all treatments exceeded the threshold of 80 kJ/kg of dry air, characterizing conditions conducive to animal heat stress [23].

The thermal profile of the transported pigs is particularly concerning, as it highlights extreme enthalpy values, which represent the thermal energy of the air. This is considered a critical factor for animal welfare during live animal transport [24]. During transport, high enthalpy conditions are widely recognized as one of the most relevant risk factors for the occurrence of ambulatory injuries and increased mortality rates upon arrival [6]. As a consequence of exposure to an environment with greater thermal energy in the air, increased rectal temperature and respiratory rate were observed in animals transported for 30 min, indicating a higher demand on the thermoregulatory system to cope with heat stress.

The behavioral results observed during transportation support this hypothesis. The “sitting” posture is commonly interpreted as an indicator of discomfort in pigs subjected to heat stress [25]. The findings of this study revealed that loads transported for 30 min exhibited a higher percentage of pigs in the “sitting” posture and a lower percentage in the “lying” posture compared to animals transported for 150 min. However, the “sitting” posture may also be associated with space limitations in the transport compartment during the trip [5]. Thus, the high stocking density adopted by the transport company in this study (290 kg/m^2^) likely negatively influenced animal welfare, including thermal discomfort, considering that a 100 kg pig emits, on average, approximately 160 W [26].

However, the observed increases in cortisol and creatine kinase levels suggest an additive effect of physiological and physical stress. Cortisol reflects the activation of the hypothalamic–pituitary–adrenal axis, while the increase in CK indicates muscle damage due to intense physical exertion [4]. Together, these indicators highlight the negative impact of the loading process on animal health and welfare. Therefore, this result can likely be attributed to the loading stage. It appears that a high stress load occurs during loading, involving significant physical effort by the animals. Previous studies corroborate and align with these findings, reporting increases in heart rate [27] and levels of salivary cortisol and blood lactate in pigs recorded by Correa et al. [28], during animal loading compared to the values observed in resting pigs. Conversely, Yu et al. [29] observed increased plasma creatine kinase (CK) levels in pigs transported for 1, 2, or 4 h, associating longer trips with elevated CK activity, suggesting a linear relationship between transport time and increased enzyme levels.

In the present study, after transportation, the highest frequency of drinking behavior was recorded in animals transported for 30 min. Furthermore, the highest frequency of agonistic behaviors was observed in animals transported for 30 min. Consequently, there was an increase in the incidence of fatigued animals and mortality rates upon arrival in loads transported for 30 min, along with a significant increase in total productive losses (Table 4). This outcome may be related to the fact that longer transport times allowed animals to recover from the stress caused by handling during loading and provided time to adapt to the social and physical environment they were exposed to during transportation [19].

While physiological stress responses and behavioral alterations observed post-transport may be influenced by handling (loading and unloading) and vehicle operation, all pre- and post-transport procedures in this study were strictly standardized (e.g., access ramps, driver training, road conditions, and sampling methods). Additionally, two drivers were used, one for each route, both highly trained and adhering to uniform operational protocols. This standardization ensured that the differences observed were genuinely due to transport effects rather than procedural inconsistencies. These results support the interpretation that shorter transport distances result in a reduced adaptation time to transport conditions (e.g., microclimate, noise, vibrations, and batch mixing), aligning with findings from previous studies. Haley et al. [30] reported that for every 50 km increase in pre-slaughter pig transport distance, the DOA rate is expected to decrease by approximately 0.81 times, with a reduced risk of DOA rates for trips exceeding 134 km. Therefore, similar to the present study, evidence suggests that depending on the magnitude of stress during loading, animals may face greater difficulty recovering their physiological state during shorter trips, particularly under semi-arid climatic conditions. Additionally, inadequate management practices during loading can result in the transport of unfit animals with pre-existing conditions. In a study conducted in Brazil, Pereira et al. [31] observed a 1.32 times higher risk of DOA in pigs that were non-ambulatory or exhibited hernias or arthritis at the time of loading. The authors also reported that of the 0.38% of pigs identified as non-ambulatory after transport, half already had this condition before loading. Thus, consistent employee training and reduced stocking density are recommended as measures to improve transport quality in semi-arid climates, especially training for loading and unloading procedures.

Based on these results, reducing stocking density to increase available space per animal can serve as a key strategy to mitigate heat stress and enhance animal welfare during transport in semi-arid regions. Lowering the number of animals per unit area reduces total metabolic heat production and humidity levels inside the trailer, leading to a more thermally comfortable environment [31]. Although reducing stocking density may increase the number of trips required, potentially impacting operational efficiency, the improved animal health and welfare outcomes, as well as lower costs associated with mortality and productive losses, justify adopting this practice. The European Union’s transport regulations recommend a maximum stocking density of 235 kg/m^2^, a guideline based on studies demonstrating that higher densities compromise resting behavior and, consequently, animal welfare. Given the complexity of these factors, further studies are needed to refine best practices for different transport scenarios, particularly under semi-arid conditions, where thermal stress is a critical challenge.

## 5. Conclusions

Shorter transport operations in the Brazilian semi-arid region posed a greater risk to animal welfare and were more susceptible to productive losses under the study conditions, likely due to the physiological and behavioral stress induced by handling, particularly during the loading process.

## Figures and Tables

**Table 1:** Ethogram for analyzing the behavior of weaned pigs.

During Transport	
Behavior	Description
Lying	The animal is in a horizontal position, with the weight of its body supported on one of its shoulders or on the sternum/belly.
Standing	The animal is in an upright position, keeping all legs straight and supporting the weight of its body on all four hooves.
Sitting	The animal keeps its front legs straight, while its rear legs are bent, keeping its hindquarters in contact with the ground.
Other posture	Any posture other than the previous ones.
**After Transport—Termination Bays**	
**Behavior**	**Description**
Drinking	The animal places its mouth around the drinker for any length of time ^1^.
Agonistic acts	The animal bites or hits another animal with its head.

^1^ A new drinking behavior was considered when the animal’s mouth was away from the drinker for at least 5 s.

**Table 2:** Means ± standard errors of air temperature, relative humidity, and enthalpy for transported loads.

Item	Transport Time		*p*-Value
	30 min	150 min	
Temperature (°C)	30.80 ± 1.03	30.46 ± 0.97	0.140
Relative Humidity (°C)	82.00 ± 2.00 ^a^	74.00 ± 1.80 ^b^	<0.001
Enthalpy (kJ/kg dry air)	88.16 ± 0.86 ^a^	81.05 ± 0.62 ^b^	<0.001

Means followed by the same letter within the same row do not differ statistically according to the Tukey–Kramer test (*p* < 0.05).

**Table 3:** Means ± standard errors of physiological variables in weaned pigs transported for 30 and 150 min.

Item	Transport Time		*p*-Value
	30 min	150 min	
Rectal temperature (°C)	39.85 ± 0.48 ^a^	39.08 ± 0.64 ^b^	0.019
Surface temperature (°C)	39.61 ± 0.69	39.58 ± 0.79	0.280
Respiratory rate (breaths/min)	98.00 ± 1.50 ^a^	90.00 ± 3.00 ^b^	0.017
Cortisol (ng/mL)	82.17 ± 1.08 ^a^	78.21 ± 1.12 ^b^	<0.001
Creatine kinase (U/L)	4140 ± 34.20 ^a^	3456 ± 32.66 ^b^	<0.001

Means followed by the same letter within the same row do not differ statistically according to the Tukey–Kramer test (*p* < 0.05).

**Table 4:** Means ± standard errors of the proportions of weaned pigs exhibiting each posture during and after transportation for 30 and 150 min, and frequency of drinking and agonistic behaviors after transport.

Behavior During Transport	Transport Time		*p*-Value
	30 min	150 min	
Standing, %	26.50 ± 14.70	25.50 ± 12.00	0.811
Lying, %	70.00 ± 12.00 ^b^	73.00 ± 10.80 ^a^	0.022
Sitting, %	3.30 ± 1.10 ^a^	1.30 ± 0.80 ^b^	0.017
Other postures, %	0.20 ± 0.60	0.20 ± 0.50	0.876
**Behavior after transport**	**Transport time**		***p*-value**
	**30 min**	**150 min**	
Standing, %	15.20 ± 6.40	15.80 ± 3.40	0.760
Lying, %	83.30 ± 7.00	82.60 ± 8.20	0.672
Sitting, %	1.20 ± 0.80	1.30 ± 0.60	0.583
Other postures, %	0.30 ± 0.50	0.30 ± 0.50	0.627
Drinking ^1^	12.20 ± 3.00 ^a^	8.00 ± 4.00 ^b^	0.018
Agonistic acts ^1^	1.80 ± 0.30 ^a^	0.40 ± 0.20 ^b^	0.043

Means followed by the same letter within the same row do not differ statistically according to the Tukey–Kramer test (*p* < 0.05). ^1^ Group of 8 animals.

**Table 5:** Means ± standard errors of the proportions of productive losses in loads transported for 30 and 150 min.

Item	Transport Time		*p*-Value
	30 min	150 min	
NANI, %	4.08 ± 0.02 ^a^	1.02 ± 0.04 ^b^	<0.001
NAI, %	2.04 ± 0.10	1.02 ± 0.04	0.137
DOA, %	6.12 ± 0.08 ^a^	2.04 ± 0.06 ^b^	0.027
Total Loss, %	12.24 ±0.06 ^a^	3.06 ± 0.05 ^b^	<0.001

Means followed by the same letter within the same row do not differ statistically according to the Tukey–Kramer test (*p* < 0.05). Non-ambulatory non-injured (NANI), non-ambulatory injured (NAI), deaths on arrival (DOA).

## Data Availability

The data used in this research are confidential.
